# Living with diabetes: rationale, study design and baseline characteristics for an Australian prospective cohort study

**DOI:** 10.1186/1471-2458-12-8

**Published:** 2012-01-05

**Authors:** Maria Donald, Jo Dower, Robert Ware, Bryan Mukandi, Sanjoti Parekh, Christopher Bain

**Affiliations:** 1 School of Population Health, University of Queensland, Brisbane, Queensland, Australia; 2 Genetics and Population Health Division, Queensland Institute of Medical Research, Brisbane, Queensland, Australia; 3 University of Queensland, Room 119, Level 2, Public Health Building, Herston, Brisbane, Qld 4006, Australia

## Abstract

**Background:**

Diabetes mellitus is a major global public health threat. In Australia, as elsewhere, it is responsible for a sizeable portion of the overall burden of disease, and significant costs. The psychological and social impact of diabetes on individuals with the disease can be severe, and if not adequately addressed, can lead to the worsening of the overall disease picture. The Living With Diabetes Study aims to contribute to a holistic understanding of the psychological and social aspects of diabetes mellitus.

**Methods/Design:**

The Living With Diabetes Study is a 5-year prospective cohort study, based in Queensland, Australia. The first wave of data, which was collected via a mailed self-report survey, was gathered in 2008, with annual collections thereafter. Measurements include: demographic, lifestyle, health and disease characteristics; quality of life (EQ-5D, ADDQoL); emotional well-being (CES-D, LOT-R, ESSI); disease self-management (PAM); and health-care utilisation and patient-assessed quality of care (PACIC). 29% of the 14,439 adults who were invited to participate in the study agreed to do so, yielding a sample size of 3,951 people.

**Discussion:**

The data collected by the Living With Diabetes Study provides a good representation of Australians with diabetes to follow over time in order to better understand the natural course of the illness. The study has potential to further illuminate, and give a comprehensive picture of the psychosocial implications of living with diabetes. Data collection is ongoing.

## Background

Diabetes mellitus currently affects about 285 million adults worldwide, and this figure is expected to rise to over 400 million adults by 2030 [1]. Based on self-reported data, the prevalence of diagnosed diabetes among Australian adults is 4.4% [2]. It is possible that the true prevalence is as much as twice that, and likely to increase further given an aging population, more sedentary lifestyles, rising rates of obesity, and a reduction in the rates of diabetes-related mortality [3-5].

The day to day management of diabetes is demanding and can take a heavy psychological and social toll, which may in turn result in poor control of blood glucose levels and an increased risk of complications [6,7]. From the patient's perspective, minimising the burden imposed by diabetes requires an approach that ensures services are integrated, accessible and affordable. They should also be patient-centred, with a strong emphasis on supporting patients' confidence and ability to effectively manage their illness [8,9]. To this end, patient reported outcomes such as quality of life and assessments of quality of care are becoming more widely used indicators of health care systems, and are now commonly considered to be critical to the evaluation of the responsiveness of health systems in meeting the needs of their users [10-12].

There is a growing literature on the interaction between various patient-reported outcomes, demographic factors, the self-management of patients with chronic illnesses, and medical outcomes [13,14]. For instance, diabetes patients with higher levels of active self-management enjoy better health outcomes [15,16]; more engaged, informed, confident, and skilled patients are more likely to perform activities that will promote their own health, and are more likely to have their health care needs met [17]. Fostering patients to take on a meaningful role in their own care is therefore central to improving quality of care and health outcomes.

There is a need to better understand the realities of living with diabetes in order to tailor adequate and appropriate medical and psychosocial interventions [18,19]. Many studies have focused on quality of life [20,21], patient activation [15,16], resource utilisation [22,23], or the clinical aspects of diabetes [24,25], but there has not been a concerted effort to simultaneously address all of these in order to gain a more holistic understanding. The Living With Diabetes Study (LWDS) described here extends the focus of previous research into diabetes beyond medical endpoints to encapsulate a broader range of outcomes that contribute to good health and improved quality of life. In particular the LWDS considers: how diabetes affects participants' quality of life, including their mental health and well-being; how satisfied people with diabetes are with the range of health services they use; how people with diabetes manage their condition; and the natural progression of diabetes over time. The findings from the LWDS will provide a more comprehensive picture of the everyday experiences of people living with diabetes and inform health policy planning and service delivery.

## Methods/Design

### Study design and sampling scheme

The LWDS is a 5-year, prospective cohort study being conducted in the state of Queensland, Australia. Data are collected via a mailed self-report questionnaire. Baseline data were collected in 2008 and follow-up measurement waves occur annually.

Participants were recruited from the National Diabetes Services Scheme (NDSS), a government initiative administered by Diabetes Australia that delivers diabetes-related products at subsidised prices to registrants. In order to register with the NDSS, an individual must receive certification of a diagnosis of diabetes from a doctor or diabetes educator. The NDSS is estimated to cover 80%-90% of the Australian population diagnosed with diabetes [26].

People were eligible to participate in the LWDS if they: were aged 18 years or older; had been diagnosed with type 1 or type 2 diabetes (gestational diabetes was excluded); had a valid Queensland postal address recorded with the NDSS; and indicated on their NDSS registration that they were interested in receiving information about opportunities to participate in research. The final criterion reduced the population available for sampling from 133,851 to 58,504.

The LWDS sampling scheme oversampled in three areas of policy interest in Queensland: an outer metropolitan area, a new suburban development and a coastal agricultural community. These areas of policy interest contain approximately 7% of potential participants. While the focus of the LWDS is on documenting the lived experience of diabetes for the entire cohort, differences between these three geographically distinct areas of interest will be provided to the local state health authority for policy and planning purposes. Where appropriate findings from the cohort are weighted to adjust for the oversampling.

The sample size was calculated based on detecting an absolute change in the percentage of participants with poor health-related quality of life of at least 2.5% between the start and the end of the study. The prevalence of poor health-related quality of life among Queenslanders with diabetes at study initiation was estimated at 30% [27]. To detect an absolute change of 2.5% or greater with 90% power and alpha = 0.05 we calculated we required 3,457 participants to remain in the study at completion. To achieve this, and assuming 2% of addresses on the database would be invalid, 40% of individuals invited to enter the study at baseline would participate, and that each year 10% of participants would leave the study, we were required to invite 14,350 eligible individuals to participate in the LWDS. All eligible individuals from the three areas of policy interest were invited to participate (45% of all invitees), the remainder of invitees were from the rest of Queensland.

### Follow-up, retention and participant tracking

In order to encourage people with diabetes to participate in the study at baseline, a multi-stage follow-up procedure was used following the initial survey package mail-out. Approximately 3 weeks after the initial mail-out, all potential participants were sent a letter designed to thank those people who had returned the survey and prompt those who had not yet returned the questionnaire. Six weeks after the initial mail-out, potential participants who had still not returned a survey were sent a replacement survey package. No further follow-up at baseline was permitted by the NDSS.

In order to minimise non-response for subsequent annual data collection waves, in addition to the thank you/reminder letter and targeted replacement survey mail-outs, targeted reminder letters are sent to those participants who have still not returned a survey 3 weeks after the replacement survey mail-out (i.e. 9 weeks after the initial mail-out). In 2010, due to concerns about the retention rate, targeted reminder telephone calls were also made to those participants who had still not returned a survey after the replacement surveys were sent out.

In order to further maximise retention of cohort participants following recruitment, a range of additional strategies and procedures are also used. For the annual follow-up data collections, all participants are sent a small incentive with the questionnaire (e.g. $1 'scratch-it' ticket in 2009 and a pen in 2010), and those who return the questionnaire go into a draw to win one of five $1,000 cash prizes. Other strategies include recording the contact details of two alternative contacts for each participant at baseline to assist with tracking; a study website and freecall 1800 number which allows people to update their contact details; biannual study newsletters for participants providing study findings as well as a reminder to update contact details; the inclusion of study synopses in relevant consumer-based organisations' newsletters; and a reminder package, which includes a postage-paid change of address card, sent 4 weeks before the annual survey mail-out.

A participant tracking system involves contacting alternative contacts for participants whose survey packages are 'returned to sender' (RTS) or who are unable to be contacted by telephone during follow-up. If no alternative contacts have been provided an online electronic phonebook is used in an attempt to track the participants. Data linkage to Australia's National Death Index (NDI) occurs annually prior to each data collection to identify deceased LWDS participants.

### Response rates

**Figure 1:**
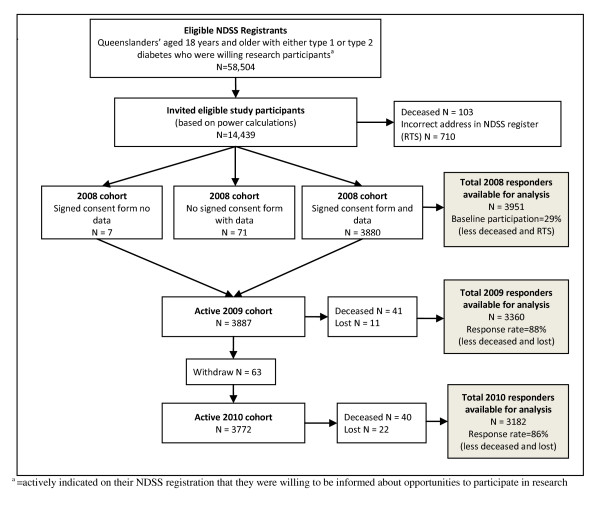
**Flow chart of study design and response rates**.

The participation rate at baseline was 29% after notified deaths and RTS were omitted (5.6%; n = 813). A study flow chart is presented in Figure 1. The retention rate in 2009 was 88% after notified deaths and RTS were omitted from calculations. This figure was 86% for the 2010 data collection.

### Participants versus non-participants

A previous analysis, using aggregated data provided by NDSS, compared the study's participants with non-participants, as well as the study's participants with all other NDSS registrants in Queensland including those who on their NDSS registration form did not consent to research participation [28]. The findings showed that participants were more likely than non-participants to be aged 50 to 69 years and to be non-indigenous Australians (see Table 1). The analyses comparing study participants with the broader population of NDSS registrants showed that the study's participants were more likely than NDSS registrants to be male, aged 50-69 years, be recently registered with the NDSS scheme and be non-indigenous Australians. In addition, there was an underrepresentation of patients with type 1 diabetes among study participants (4.8%) compared to NDSS registrants (14.5%).

**Table 1:** Comparison between LWDS participants and non-participants (adapted from David, Ware, Donald et al., 2011)

	Participants	Non-participants	OR (95% CI)
	N = 3,951	N = 10,488	
**Sex**			
Male	2,176 (55.1%)	5,885 (56.1%)	1
Female	1,775 (44.9%)	4,603 (43.9%)	1.01 (0.93-1.10)
**Age**			
18-49	618 (15.6%)	2,246 (21.4%)	0.67 (0.60-0.75)
50-69	2,375 (60.1%)	5,649 (53.9%)	1
70+	958 (24.3%)	2,593 (24.7%)	0.88 (0.80-0.97)
**Diabetes Status**			
Type 2 No Insulin	3,023 (76.5%)	8,024 (76.5%)	1
Type 2 Insulin	738 (18.7%)	1,986 (18.9%)	0.97 (0.87-1.08)
Type 1 Insulin	190 (4.8%)	478 (4.6%)	1.11 (0.91-1.34)
**Registration Year**			
2001-2003	1,303 (38.0%)	3,422 (37.0%)	1
2004-2005	805 (23.4%)	2,239 (24.2%)	0.96 (0.85-1.07)
2006-2008	1,325 (38.6%)	3,580 (38.8%)	1.01 (0.92-1.12)
**Socioeconomic status**			
Low	830 (21.0%)	2,491 (23.8%)	1
Middle	1,543 (39.1%)	3,883 (37.1%)	1.13 (1.01-1.27)
High	1,572 (39.9%)	4,100 (39.1%)	1.11 (0.99-1.24)
**Indigenous**			
No	3,838 (97.2%)	9,969 (95.1%)	1
Yes	113 (2.8%)	519 (4.9%)	0.57 (0.45-0.71)

### Measures and instruments

The study collects information on a range of issues, including the primary outcomes of interest--quality of life and quality of care. Clinical outcomes such as the onset of diabetes complications and HbA1c are also measured. An overview of the components of the survey is provided in Table 2. Information relating to 347 primary variables was collected in the 2008 LWDS questionnaire. A copy of the questionnaire is available from the authors on request or can be downloaded from the study's website at http://www.lwds.org.au.

**Table 2:** Summary of main variables collected

Section	Examples of Variables	Measures and Standardised Scales
**A: Disease-related factors**	Type of diabetes and Disease duration Glycaemic control, Treatment type, Diabetes Complications & Co-morbidity	Diabetes type verified using NDSS data
**B: Health and Lifestyle**	Nutrition Smoking status Alcohol consumption Physical activity Height, Weight, Sleep patterns (2010 only)	Dietary Guidelines for Australian Adults [29] National Drug Strategy Household Survey [30] Frequency-quantity measure^# ^& National Health and Medical Research Council Guidelines [31]. Active Australia Survey [32] & National Physical Activity Guidelines for Australians [33] Body Mass Index adapted from WHO [34]
**C: Quality of Life**	Health related quality of life Diabetes-specific quality of life	Euroqol EQ-5D [35] Audit of Diabetes Dependant Quality of Life (ADDQoL) [36,37]
**D: Disease management**	Self-management Compliance with providers recommendations Disease management resources (2010 only)	Patient Activation Measure (PAM) [38] Summary of Diabetes Self-Care Activities (Modified) [39] Chronic Illness Resources Survey (CIRS) [40]
**E: Health care utilisation**	Primary care visits Allied health visits Emergency department visits Hospitalisations and reasons	
**F: Quality of care**	Patient-assessed quality of care Satisfaction with care (access, coordination) Practitioner compliance with guidelines	Patient Assessment of Chronic Illness Care (PACIC) [41] Australian Diabetes Management in General Practice guidelines [42]
**G: Emotional well-being**	Depressive symptomatology Stressful life events Optimism (2009, 2010) Social Support (2009, 2010)	Centre for Epidemiological Studies: Depression Scale (CES-D) [43] Life Orientation Test - Revised (LOT-R) [44] ENRICHED Social Support Inventory (ESSI) [45]
**H: Socio-demographics**	Age, Sex, Marital status, Ethnicity, Employment status, Educational status & Household income Private health insurance; Health concession cards, Out of pocket health costs	Derived from Australia's National Health Survey questions [46]

^# ^The frequency-quantity method used to measure alcohol consumption in the LWDS is a relatively conservative estimate of high risk alcohol consumption [47]

The NDI will be used to identify premature deaths, and cause of death data will be accessed at the completion of the study. In addition, a proposed data linkage to Australia's Medical Benefits Scheme (MBS) and Pharmaceutical Benefits Scheme (PBS) has also gained ethics approval and will provide information about individual participant's use of government subsidised health services and medications. Access to these administrative data will not only provide information to verify self-report health care utilisation data, but will also allow for more detailed examination of the timing of services and health outcomes.

### Main outcomes of interest

#### Health-related quality of life

The EQ-5D, developed by the EuroQoL Group, is widely used in clinical trials, observational studies and health surveys, and has been translated into most major languages [35,48]. The measure includes a descriptive system comprising five dimensions and a visual analogue scale. The dimensions measured are: mobility, self-care, usual activities, pain/discomfort and anxiety/depression. To complete the visual analogue scale respondents are asked to rate their current health status on a scale from 0 to 100, where 0 is the worst imaginable health and 100 is the best imaginable health. The EQ-5D also provides a preference-based utility index where responses are converted to a single weighted score. This gives a possible 243 health states, 245 when unconscious and dead are included. An EQ-5D score of 1 corresponds to perfect health; 0 to indifference between death and living; and, any numbers less than 0 to a state where death is preferred.

#### Diabetes-specific quality of life

The ADDQoL consists of two overview items; one measures generic overall quality of life and the second measures the specific impact of diabetes on quality of life. A further 19 domains concerned with the impact of diabetes on specific aspects of life are also measured. Participants are asked to rate the impact of diabetes on each domain and the importance of the domain for their quality of life. These two scores are then multiplied to yield a weighted impact score for each domain (range -9 to +3). Finally, an average weighted impact score is then calculated for the entire scale.

#### Quality of care

Respondents completed the Patient Assessment of Chronic Illness Care (PACIC), which measures the extent to which patients report receiving care that is consistent with the dimensions of the Chronic Care Model (CCM) [49]. The PACIC consists of 20 items with each item scored using a five point scale, ranging from 1 being none of the time to 5 being always. The items aggregate into five sub-scales that align with the dimensions of the CCM: patient activation, delivery system design/decision support, goal setting/tailoring, problem solving/contextual, and follow-up/coordination. Patient activation assesses the extent to which the patient was motivated and supported by the physician to initiate changes. Delivery system design/decision support assesses the degree to which the patient was supported (e.g. by information sheets), and how satisfied the patient was with the organisation of that care. Goal setting/tailoring assesses to what extent general instructions and suggestions were adapted to the patient's personal situation. Problem solving/contextual addresses how the physician dealt with problems which interfered with achieving predefined goals. Finally, follow-up/coordination addresses how frequently and consistently the care process was followed-up. Each sub-scale is scored by averaging across the items within that sub-scale, with an overall PACIC score obtained by averaging across all 20 items. Higher scores indicate higher quality care.

To provide another measure of quality of care, participants completed a series of purpose-designed quality indicators based on specific aspects of care recommended in the Australian Diabetes Management in General Practice guidelines [42], by indicating whether a member of their medical team had undertaken the care activity in the preceding 12 months.

### Ethics

Ethics approval for the study was granted by the University of Queensland's Behavioural and Social Sciences Ethical Review Committee. Written informed consent was obtained from all individuals followed over time. Consent forms are removed from returned surveys and stored in a locked filing cabinet.

### Data management and statistical analyses

After entry, data is both manually and statistically checked during the data-cleaning process. Data are verified against the returned surveys when necessary. Electronic scanned copies of all original surveys are kept on file in a password-protected data file while hard copies are destroyed.

Initially, descriptive statistics are calculated for all variables. Where appropriate proportions are weighted to adjust for the oversampling, and unweighted sample sizes and weighted percentages are reported. Change over the follow-up period will be assessed using generalised estimating equations (GEE), which will allow for the best use of all data collected as GEEs do not require balanced data sets. Using GEEs will allow the data of all individual participants to be included in analyses, irrespective of the level of questionnaire completion.

The exact final method of analysis will be outcome-dependent. However, as a rule all associations will be assessed in the following stepwise manner: (a) univariate analysis: only adding the specific independent variable to the model; (b) a multivariate model to establish the significant associations; (c) adjusting for age, gender and socio-economic indicators to explore potential confounding factors; and (d) the addition of potential effect-modifiers by using interaction terms wherever necessary.

### Baseline characteristics of the sample

Baseline socio-demographic characteristics of the sample are presented in Table 3 for the purpose of assessing the potential generalisability of the findings to Australians with diabetes. Fifty-five percent of the research participants were male. Participants' age ranged from 18 to 94 years. The overall mean age was 61.4 years (SD = 12.1). Educational status varied; 13.4% of participants had completed university study, while almost half (46.5%) reported education to grade 10 (i.e. junior certificate) or lower. A large proportion of the sample (44.5%) was retired. Only 1.8% of the study population reported being of Indigenous origin. Almost two thirds of participants live in households earning less than $60,000 (AUD) per annum, while 30.6% of LWDS households earned less than $20,000 (AUD) per annum. 62.0% of LWDS participants reported that they hold a health care card which provides access to subsidised health care.

**Table 3:** Baseline socio-demographic characteristics

Socio-demographic characteristic	All Persons n = 3951	%
Gender		
Male	2175	55.1%
Female	1776	44.9%
Age		
18-44 year	343	8.7%
45-59 year	1204	30.5%
60-74 year	1921	48.6%
75 + year	483	12.2%
Education Level		
University degree	516	13.4%
Certificate/diploma/trade	1044	27.2%
Senior high school	495	12.9%
Year 10 and below	1789	46.5%
Employment Status		
Full time/part time/self-employed	1521	39.1%
Home duties/carer/volunteer	241	6.2%
Unemployed (but able to work)	94	2.4%
Retired	1729	44.5%
Unable to work	301	7.8%
Married or living as married	2766	70.8%
English speaking background	3750	98.2%
Indigenous Australian	70	1.8%
Household annual income < $60,000(AUD)	2540	73.7%
Health concession cards (yes)	2394	62.0%

Baseline medical and lifestyle characteristics of the LWDS sample are summarised in Table 4. The median duration of time since receiving a diagnosis of diabetes was 5 years and the mean was 6 years (range: less than 1 year to 50 years). Overall, 83% of participants reported being diagnosed within the past 8 years, with more than half (69.7%) managing their diabetes with oral hypoglycaemic drugs. Approximately one out of five participants was insulin requiring. Erectile dysfunction was the most commonly reported diabetes complication among men (41.4%), followed by diabetes related eye disease (e.g. retinopathy, cataracts, glaucoma), which was the highest among women. Overall, one in ten LWDS participants smoked daily. More than four out of five participants were overweight, with half being obese or morbidly obese.

**Table 4:** Baseline medical and lifestyle factors

Medical or lifestyle factor	All Persons	Males	Females	*p*-value
**Medical**				
Type of diabetes				
Type 1	4.8%	4.5%	5.2%	0.256
Type 2	95.2%	95.5%	94.8%	
Length of diagnosis				
2 years or less	25.5%	25.4%	25.6%	0.298
3 to 8 years ago	57.5%	56.7%	58.3%	
More than 8 years ago	17.0%	17.8%	16.1%	
Diet only treated diabetes	4.1%	3.8%%	4.4%	0.372
Current oral hypoglycaemic agents	69.7%	71.8%	67.7%	0.005
Insulin requiring	21.6%	22.7%	20.2%	0.055
HbA1c Levels				
7.0% or lower	48.7%	48.2%	49.2%	
7.1%-8.0%	21.7%	22.4%	20.8%	0.070
Over 8.0%	14.3%	15.1%	13.3%	
Don't know	15.4%	14.3%	16.7%	
Lipid-lowering agents	52.1%	52.8%	51.0%	0.224
Antihypertensive therapy	57.3%	57.9%	56.6%	0.412
Current depression	38.5%	35.7%	41.8%	< 0.001
**Diabetes Complications**				
Eye disease	23.3%	23.7%	22.8%	0.500
Kidney disease	6.2%	6.8%	5.5%	0.090
Neuropathy	8.8%	9.4%	8.2%	0.192
Erectile dysfunction	-	41.4%	-	-
Foot ulcers	2.1%	2.6%	1.5%	0.017
Heart disease^a^	15.4%	19.4%	10.6%	< 0.001
Stroke or transient ischaemic attack	5.1%	6.0%	3.9%	0.003
**Behavioural lifestyle**				
Inadequate fruit consumption	39.5%	43.4%	34.8%	< 0.001
Inadequate vegetable consumption	87.2%	90.1%	83.6%	< 0.001
Insufficient physical activity^b^	50.7%	45.2%	57.3%	< 0.001
Current smokers	10.5%	10.7%	10.2%	0.619
Risky alcohol consumers	6.6%	8.5%	4.6%	< 0.001
BMI (mean = 31.1 ± 6.9 kg/m^2^)				
Underweight	0.5%	0.2%	0.9%	< 0.001
Normal	16.4%	15.7%	17.3%	
Overweight	33.3%	39.2%	25.9%	
Obese or morbidly obese	49.7%	44.9%	55.8%	

^a ^includes heart disease, angina, heart attack, irregular heart rhythm, missed heart beats or blocked artery in the heart

^b ^insufficient for health benefit is defined as less than 150 min of at least moderate-intensity physical activity over at least five sessions in the previous week (18-75 years only)

Given established differences between males and females on several factors with likely implications for intervention, Table 4 also presents medical and lifestyle characteristics stratified by gender. Sub-group analysis by gender showed no significant differences for the diabetes-specific characteristics such as type of diabetes, length of diagnosis or HbA1c levels. However, gender specific differences were observed for several diabetes complications: including foot ulcers, cardiovascular disease and stroke. In addition, females were more likely than males to report co-morbid depressive symptomatology. With the exception of current smokers, statistically significant gender differences were observed for each of the health risk behaviours, including body mass index (BMI).

## Discussion

This paper describes the study rationale and procedures for the LWDS and reports the baseline characteristics of a cohort of 3951 people with diabetes. The approach of the LWDS differs from other national and international studies of diabetes in its focus on examining the natural trajectory of diabetes and its treatment from a psychosocial perspective. It is hoped that this will lead to a greater understanding of how to improve the life and quality of care of people with diabetes. The large cohort will allow for the undertaking of multivariate statistical analyses, and the longitudinal nature of the data enables the investigation of temporal effects.

Wherever possible standardised scales and questions were used in the LWDS questionnaire to measure the domains of interest. We relied on previous health services research and psychosocial research to select measures sensitive to change and with adequate face, content and construct validity. The importance of distinguishing between health-related quality of life and disease-specific quality of life has been highlighted by previous researchers [11,50]. On this basis, both are measured in the LWDS. Health-related quality of life measures a patient's symptoms and functioning. The EQ-5D is one of the most widely used preference-based measures of health-related quality of life [51]. Disease-specific quality of life, which captures the broader multidimensional, subjective and dynamic features of quality of life, relates more specifically to a person's perception of how a specific disease has impacted on their life [52]. Disease-specific measurement instruments include those aspects of life considered to be the most important by patients and clinicians resulting in a more detailed assessment of the issues and concerns relevant to the specific disease, its treatments and complications [13,37,53]. Several recent reviews of diabetes-specific quality of life instruments conclude that there is good evidence that the ADDQoL, used in the LWDS, is a reliable measure of disease-specific quality of life with good face and content validity [52-55]. A recent assessment of 37 measures of patient-assessed quality of care designed for use with people with chronic illness found the PACIC, used in the LWDS, to be the most appropriate as determined by its psychometric properties and perceived applicability and relevance [41]. The inclusion of these self-reported patient assessments of quality of life and quality of care will allow for the reliable assessment of the impact that the progression of diabetes has on these important health and well-being outcomes.

The LWDS study has the inherent limitations of self-report surveys. The reliability and validity of self-report health measures varies across behaviours and outcomes. Self-reported health service utilisation data is subject to recall bias and underreporting, especially for older adults and frequent users of primary care [56,57]. Cross-referencing the self-report service utilisation data with MBS data will improve the reliability and validity of this information. Self-report data on health information, such as co-morbidities, has been found to be of variable quality, but is generally satisfactory for well-known conditions [58,59]. Previous research has found a positive, albeit weak, correlation between self-reported HbA1c values and medical record data [60]. Self-report of treatment types including oral agents and insulin use are generally valid [61].

While recruitment at baseline was slightly lower than anticipated, it is similar to that of other studies of this nature [62] and is consistent with research showing that participation rates in large cohort studies appear to be decreasing. It is estimated that rates have declined from about 80% to 30% or 40% over the past several decades [63]. Effective participant retention is vital to the success of the LWDS and we have instituted an anti-attrition strategy to ensure as many cohort members as possible remain in the study. Past research suggests that the attrition in follow-up for postal surveys can be decreased by the provision of a monetary incentive such as a lottery ticket or a prize [64], with the magnitude less important than an incentive *per se *[65]. Although losses to follow-up are inevitable, the study's comprehensive retention strategy to date has been successful in limiting participant drop-out. However, it is possible that those individuals whose health deteriorates more markedly over time will discontinue study participation, biasing findings towards healthier participants.

Use of a national disease register with high coverage of the target population in the recruitment process has been effective for enrolling a large representative sample of people with diabetes, covering a broad range of socio-demographic and clinical characteristics. Specifically, the proportion of males was consistent with estimates provided by Australia's National Health Survey that 56% of Australians with diabetes are male [2]. The large proportion of LWDS participants with low levels of educational attainment is not unexpected given the older age distribution of the sample. Similarly, it is unsurprising that a large proportion of the sample (44.3%) was retired and one in 15 reported being unable to work.

In 2005-06 the average gross annual household income in Australia was around $68,000 (AUD) [66]. While it is difficult to make direct comparisons based on the income brackets used in the LWDS, the finding that 73.7% of participants live in households earning less than $60,000 (AUD) per annum indicates that overall the sample are more economically disadvantaged than the general population of Australians. This differential is consistent with what would be expected given the previously reported relationship between low socio-economic status and prevalence of diabetes [67,68] as well as the high levels of retirement among the LWDS participants.

A similar pattern holds for health concession cards. In Australia low income is one of the criteria for eligibility for a government health concession card. Possession of the card can significantly reduce the out-of-pocket expenses for consumers through subsidised medical care, hospital treatment and some medications. Australia's 2004-05 National Health Survey reported 35% of persons 15 years and over were covered by a government health card, a figure considerably lower than the 62.0% of LWDS participants reporting that they hold a health care card. The higher LWDS proportion will most likely be accounted for by the older age and poorer health status of people with diabetes.

Another area in which the study is not representative of the general population relates to the proportion of Indigenous Australians. Research shows that diabetes is more common among Indigenous Australians [69] than among their non-Indigenous counterparts. However, with census data showing that 2.5% of the Australian population report an Indigenous background [70] and only 1.8% of the study population reporting that they are of Indigenous origin the LWDS cannot be generalised to this population.

Less than one in 20 LWDS participants relied on diet alone as the treatment pathway for their diabetes. Previous researchers have suggested that people managing their diabetes through diet and exercise alone may not have a high level of need for the NDSS's services and would therefore be less likely to register with the scheme [26]. Our findings support this assumption. Less than 5% of the LWDS sample reported having a diagnosis of type 1 diabetes. The true prevalence of type 1 diabetes in Australia is estimated to be approximately 10% [2]. The underrepresentation of patients with type 1 diabetes is attributed to the lower likelihood of NDSS registrants with type 1 diabetes consenting to participate in research as there is not systematic updating of research consent status at the age of 18 among those registered as a child [28]. We acknowledge this as a weakness of the LWDS with implications for the generalisability of the study to people with type 1 diabetes. Future analyses of the data will stratify by diabetes type.

On the other hand, the diabetes complications reported by the LWDS participants at baseline were very much in keeping with the available Australian statistics for the Australian diabetic population as a whole [71]. For example, 2.1% of respondents reported having foot ulcers, with the available comparable figure for the entire Australian diabetic population also 2.1%. Additionally, 6.2% of the sample, compared to 6.3% of the Australian diabetic population, reported kidney disease; 5.1% of the sample compared to 5.0% of the Australian diabetic population reported having had a stroke; 8.8% of the sample versus 8.6% of the Australian diabetic population reported neuropathy; and 41.4% of men on the study reported erectile dysfunction as a complication, while 30.2% of the general male diabetic population had the same complaint. The discrepancy for erectile dysfunction may stem from the differing data collection methods used to obtain the estimates.

Lifestyle changes constitute an important aspect of the management of diabetes, in particular type 2 diabetes. For example, obesity in those with type 2 diabetes complicates management of the disease by increasing insulin resistance and with that, blood glucose concentrations. This however, is reversible, such that weight loss of just 5% of body weight may improve insulin sensitivity and glycaemic control [72,73]. Research undertaken with US adults found that those with diabetes were more likely to be physically inactive (61%) than those without diabetes (42%) [74]. Recent comparable physical activity rates for the Australian general population are not available. We can however compare the LWDS with the Australian Diabetes, Obesity and Lifestyle Study (AusDiab) conducted in1999 -2000 which found that 59.3% of Australians with diabetes were insufficiently active [75], which is higher than the 50.7% found for the LWDS sample. Recent suggestions of a trend towards increased levels of physical activity among Australians may explain the difference [76]. It is well established that on average people with diabetes are more likely to be overweight than people without diabetes. Both the LWDS and the AusDiab study [71] found that approximately four out of five diabetic patients are overweight or obese. The baseline estimates for health risk behaviours for participants of the LWDS suggest that there is considerable scope for behavioural lifestyle modification among Australians with diabetes.

The wide range of information collected from the LWDS participants allows for an in-depth exploration of the multidimensional nature of diabetes. The availability of longitudinal data allows the LWDS to contribute towards a deeper understanding of the dynamics of living with diabetes; and to build complex psychosocial models of the determinants of disease progression, quality of life and models of patients' assessments of the quality of their care. This will allow for the identification of key targets for intervention strategies, and will contribute to public health policy, especially as it relates to resource allocation and planning.

## Competing interests

MD, JD, RW, BM and SP all had financial support from Queensland Health.

## Authors' contributions

MD made a substantial contribution to the conception and design of the study as well as interpretation of the data and was involved in drafting the manuscript. JD contributed to the interpretation of the data and was involved in drafting the manuscript. RW supervised the statistical analyses and was involved in drafting the manuscript. BM contributed to interpretation of the data and was involved in drafting the manuscript. SP performed the statistical analysis and was involved in drafting the manuscript. CB contributed to the conception of the study and was involved in revising the intellectual content of the manuscript. All authors read and approved the final manuscript.

## Pre-publication history

The pre-publication history for this paper can be accessed here:

http://www.biomedcentral.com/1471-2458/12/8/prepub
